# Application of *C. elegans* cancer screening test for the detection of pancreatic tumor in genetically engineered mice

**DOI:** 10.18632/oncotarget.27124

**Published:** 2019-09-10

**Authors:** Yuji Ueda, Koichi Kawamoto, Masamitsu Konno, Kozo Noguchi, Satoru Kaifuchi, Taroh Satoh, Hidetoshi Eguchi, Yuichiro Doki, Takaaki Hirotsu, Masaki Mori, Hideshi Ishii

**Affiliations:** ^1^ Department of Gastroenterological Surgery, Graduate School of Medicine, Osaka University, Osaka 565-0871, Japan; ^2^ Department of Medical Data Science, Graduate School of Medicine, Osaka University, Osaka 565-0871, Japan; ^3^ Department of Frontier Science for Cancer and Chemotherapy, Graduate School of Medicine, Osaka University, Osaka 565-0871, Japan; ^4^ Hirotsu Bioscience Co., Ltd., Tokyo 107-0062, Japan; ^5^ Department of Biology, Graduate School of Science, Kyushu University, Fukuoka 819-0395, Japan

**Keywords:** early detection, pancreatic cancer, C. elegans, genetically-engineered mice

## Abstract

Pancreatic ductal adenocarcinoma (PDAC) exhibits a very early onset of metastasis. Thus, early detection and treatment are pivotal to successful eradication of pancreatic cancers. Economical and non-invasive cancer screening systems is indispensable for this purpose. Previously our group developed a novel method to detect various kinds of human cancer using nematode *Caenorhabditis elegans (C. elegans)* that respond to cancer odor in urine; however, whether this method is useful for non-human species remains to be understood. In this study, we examined its effectiveness in the detection of murine pancreatic tumor spontaneously generated in genetically-engineered mice. We generated pancreas-specific *Kras*^G12D^ and/or *c-Met* deletion mutant mice and measured the probability of spontaneous tumor generation in these mice. The chemotactic indexes of *C. elegans* to the urine samples of these mutant mice were measured. As previously described, oncogenic *Kras^G12D^* was necessary to induce pancreatic intraepithelial neoplasia in this mouse model, while c*-Met* mutation did not show further effect. The chemotactic analysis indicated that *C. elegans* avoids urine of healthy recipient mice, while they tended to be attracted to urine of mice with *Kras^G12D^*. Our study demonstrated that *C. elegans* can recognize the odor of pancreatic cancer in urine of *Kras^G12D^* model mouse, suggesting the similarity of cancer odor between species. Our result facilitates further studies on mechanism of cancer detection by *C. elegans*.

## INTRODUCTION

Pancreatic ductal adenocarcinoma (PDAC) is one of the most lethal cancers, and the fourth most common cause of cancer death across the world [1–2]. Complete surgical resection is the only way for long-term survival of the patients of PDAC. However, 5-year-survival rate of curative pancreatic cancer has not improved substantially in the past 30 years [1–3]. One reason why pancreatic cancer has a dismal prognosis may be asymptomatic nature of the disease. Many of PDAC patients have metastases at the time of diagnosis [2]. Thus, early-stage diagnosis of PDAC is definitely important to improve its prognosis.

Currently, carcinoembryonic antigen (CEA) and carbohydrate antigen 19-9 (CA19-9) are ones of the most established biomarkers used for the detection and management of pancreatic cancer [4]. However, sensitivity and specificity of these markers are not good enough for effectively screening pancreatic cancer in clinical practice. Thus, identification of novel biomarker for the detection of early stage of pancreatic cancer is indispensable to improve the survival of pancreatic cancer patients.

Recent progress in the study of genetically-engineered mouse model (GEMM) revealed the process of pancreatic tumorigenesis and enabled us to detect minimal change in early stage of PDAC [5]. It was reported that epithelial-to-mesenchymal transition (EMT) and dissemination precede pancreatic tumor formation in spontaneous tumor mice model [6, 7]. Nearly all PDAC and a high percentage of early pancreatic intraepithelial neoplasia (PanIN) lesions have KRAS mutations. It was reported that seeding and propagation of untransformed mouse mammary cells having Kras mutation in the lung [8]. The human acinar-to-ductal metaplasia (ADM)-to-PanIN-to-PDAC progression has been recapitulated using GEMMs [9, 10].

*C. elegans* has a highly developed chemosensory system and can detect a wide variety of volatile and water-soluble chemicals associated with food, danger, or other animals. Because of its short lifespan and hermaphroditic nature, *C. elegans* is easy to be maintained in laboratories and therefore widely used as genetic model in research. Previously, we found that *C. elegans* can detect the odor of cancer in urine of cancer patients [11, 12]. Based on this finding, we developed a novel and highly accurate cancer detection system, Nematode Scent Detection Test (NSDT) that is also economical, painless, rapid and convenient. In our report among 20 cancer patients tested, six cases were early stage cancer (stage 0 or 1), suggesting that NSDT can be used for screening of early cancer. However, our data contained only one patient with pancreatic cancer, which was at advanced stage. Thus, the effectiveness of *C. elegans* to detect early stage of pancreatic cancer has not been fully analyzed.

In mice, various kinds of strains that develop pancreatic tumor have been reported. Therefore, we decided to assess the possibility of NSDT to detect pancreatic tumor using mouse model. Oncogene Kras plays a role in the generation of pancreatic cancer, and its activation is observed in >95% cases of PDAC patients [2]. Kras^G12D^ point mutation allele is commonly associate with human cancer and leads to a constitutive active form of Kras. On the other hand, c-Met is involved in the chemotherapy resistant phonotype of pancreatic cancer [13, 14]. Therefore, we have generated mutation of Kras in pancreas specific manner in mice, which was compared with pancreas specific deletion of c-Met, as described [14].

## RESULTS

### Tumor formation in genetically-engineered model mice

**Figure 1:**
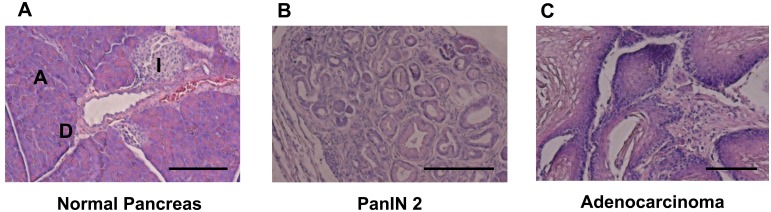
HE staining for pancreatic tissue. (**A**) Normal pancreatic tissue in LSL-KRAS^G12D^ negative and c-Met Wt mouse. Scale bar, 100μm; PanIN, pancreatic intraepithelial neoplasia; A, acinar cell; I, islet. (**B**) PanIN 2 Pancreatic Tissue in LSL-KRAS^G12D^ positive and c-Met Wt mouse. (**C**) Adenocarcinoma Pancreatic Tissue in LSL-KRAS^G12D^ positive and c-Met WT mouse.

We used a Cre-loxP-based mouse model of PDAC to assess the possibility of *C. elegans* to detect the putative odours from tumor-bearing mice. First, we assessed the tumorigenicity of oncogenic *KRas^G12D^* mice in which *Kras^G12D^* oncogene was specifically introduced in pancreas. As shown in Figure 1, 3 out of 12 (25%) *Kras^G12D^* mice have developed pancreatic tumor. Then we crossed *Kras^G12D^* mice with *c-Met* mutant mice to obtain *KRas^G12D^/c-Met* double mutant mice, and assessed the tumorigenicity. 8 out of 13 (61.5%) *KRas^G12D^/c-Met* mice have developed pancreatic tumor (Figure 1). The occurrence of tumors was not affected by *c-Met* deletion. These results demonstrated that spontaneous tumor was observed only in *Kras^G12D^* –bearing mice, suggesting that *Kras^G12D^* mutation is necessary for the development of pancreatic tumor [13–15] (Table 1). We then used urines from these mice for the assessment of cancer detection by *C. elegans*.

**Table 1:** Genotype for mouse harvested urine

Type	*Pdx1-Cre*	*LSL-KRAS*^G12D^	c-Met	Gender	Days
1a	Tg	Negative	Wt	F	249
1b	Tg	Negative	Wt	F	364
1	Tg	Negative	Wt	F	273
2	Tg	Positive	Wt	F	244
2	Tg	Positive	Wt	M	273
2	Tg	Positive	Wt	M	383
3	Tg	Negative	flox/flox	M	290
3	Tg	Negative	flox/flox	M	308
3	Tg	Negative	flox/flox	M	307
3	Tg	Negative	flox/flox	M	326
3	Tg	Negative	flox/flox	M	326
3	Tg	Negative	flox/flox	M	311
3	Tg	Negative	flox/flox	M	318
3	Tg	Negative	flox/flox	F	318
3	Tg	Negative	flox/flox	F	314
4	Tg	Positive	flox/flox	M	286
4	Tg	Positive	flox/flox	M	290
4	Tg	Positive	flox/flox	F	332
4	Tg	Positive	flox/flox	F	318

(Tg: transgenic mouse, F: female, M: male).

### Detection of tumor in mice by *C. elegans* scent

**Figure 2:**
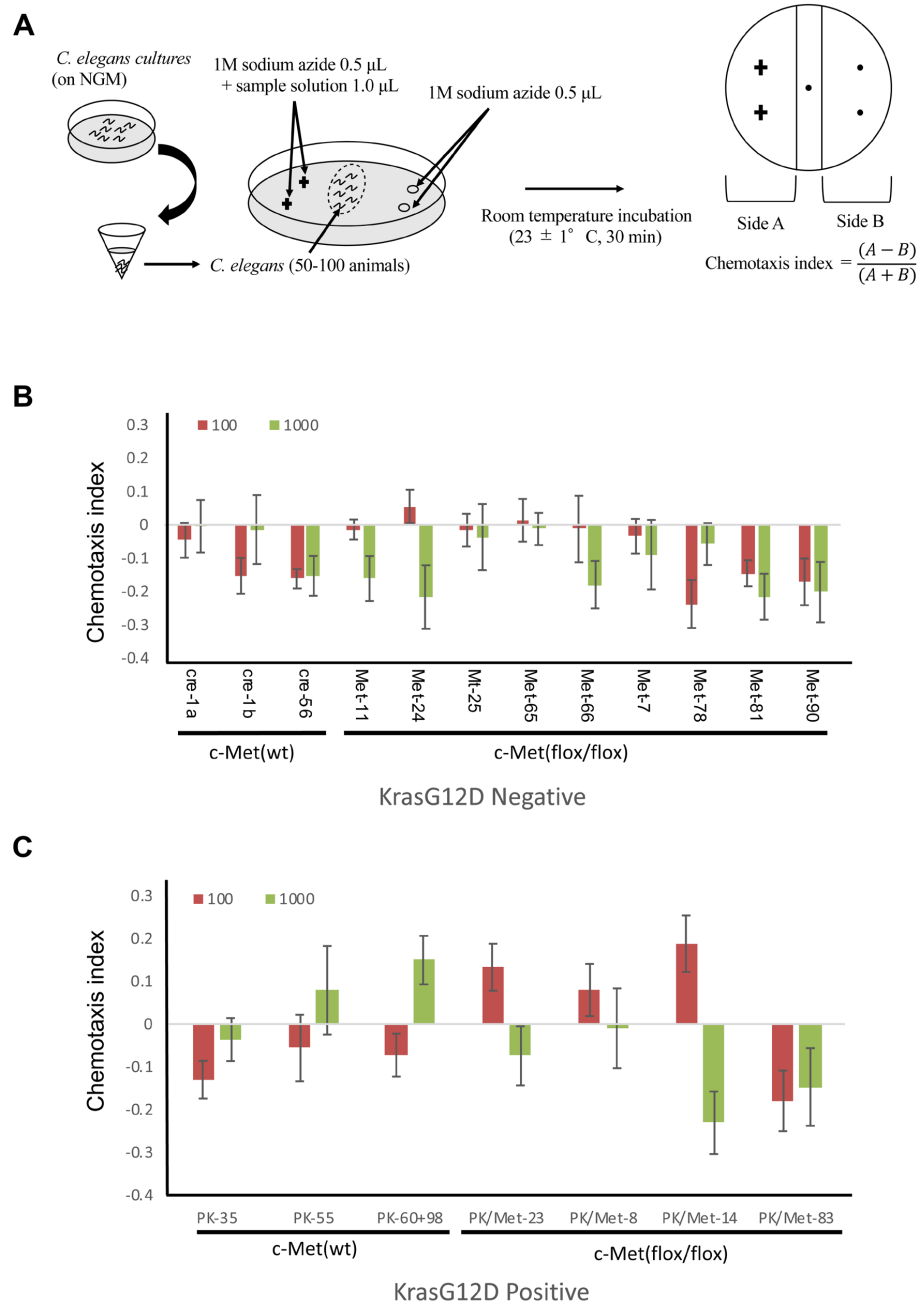
Detection of tumor in mice by *C. elegans* scent. (**A**) The method for chemotaxis assay using *C. elegans*. (**B**, **C**) The results for chemotaxis assay using *C. elegans*. Red bars mean samples were diluted at 10^2^ before analysis. Green bars mean samples were diluted at 10^3^ before analysis. Standard deviation was calculated using excel.

To test the possibility that *C. elegans* can detect odors of tumor generated in these mouse models, we performed the chemotaxis assay (Figure 2A). Our previous study demonstrated that chemotactic response of *C. elegans* to serum was not significantly different between healthy volunteers and cancer patients at any concentrations. This may be due to the presence of other odorants or molecules in the serum that mask the smell of tumor-derived molecules. Therefore, in the current mouse study, we used urine to confirm the result of human study. As the urinary concentration of mice is higher than that of human, samples were diluted at 10^−2^ and 10^−3^ before analysis [16]. Although the magnitudes of chemotactic responses were less than human cases, *C. elegans* showed avoidance against urine from healthy control littermates (Figure 2B, 2C). c-Met deletion exhibited only minimal effects on these chemotactic responses (Figure 2B, 2C). *C. elegans* showed very weak attraction to only 2 out of 9 urine samples collected from c-Met mutant mice at 10^−3^ concentration. In contrast, the chemotactic index of *C. elegans* to urine from *Kras^G12D^* mutation mice was significantly higher compared to urine of *Kras^G12D^*-negative control mice (Figure 2B, 2C). Five out of 7 urine samples from *Kras*^G12D^-positive mice attracted *C. elegans* at either 10^−3^ or 10^−4^ concentration, indicating that the sensitivity of this analysis to detect tumor is 71.4%. On the contrary, 2 out of 12 *Kras*^G12D^-negative mice attracted *C. elegans,* showing that the specificity of this analysis is 83.3%. These results suggest that volatile odor would be present only in mice with *Kras*^G12D^ and suggest the possibility that the molecule would be downstream of *Kras^G12D^* gene. Given that *Kras* gene controls the metabolic flux of glutamine in pancreatic cancer cells, we are interested in whether any detectable metabolites might influence on the behaviors of *C. elegans* [12].

## DISCUSSION

Identification of volatile biomarkers for disease diagnosis, including various kinds of cancers, is an area of great interest and promise. Originally, it was reported that trained dogs can discriminate odor of cancer patients from healthy control. Several studies demonstrated that dogs can detect odor of various cancers including colon, melanoma, bladder, lung, breast and ovarian cancer in patients’ urine or breath. However, considering the time and the cost required for dog education, it may be difficult to introduce canine scent detection into clinical practices. We have previously reported that *C. elegans* can effectively detect the odor of cancer in human urine [12]. In the present study, we demonstrated that *C. elegans* can also distinguish the odor of genetically-engineered mice from control siblings. The magnitude of chemotactic responses of *C. elegans* for murine urine (−0.25 to 0.2) was lower compared to that for human samples (−0.3 to 0.4). In addition, sensitivity (71.4%) as well as specificity (83.3%) of the analysis are not as high as for human samples (95.8% and 95.0%, respectively).

The reason of this difference may be the specie specificity of cancer odorants. The further study will be necessary to confirm this hypothesis.

The lines of our studies showed that *C. elegans* has a potential to detect volatile components in urine from cancer bearing conditions, although the molecular characteristics of the components have not been identified. Moreover, we demonstrated in this study that *C. elegans* has potential to detect cancer odors across species, both for human and murine. Limitation of current study might that we did not directly compared human and murine samples.

Our next project is to reveal the effectiveness of this system on clinical samples of various kinds of progression of PDAC patients.

The progression of PanIN to PDAC also meant that this mouse model offers the opportunity to identify precancerous lesion. Pancreatic development is initially associated with the activation of pancreatic and duodenal homeobox 1 (Pdx1). Previously, in mice bearing Pdx-1-Cre; *Kras^G12D^*; p53^fl/+^; Rosa^YFP^ (PKCY) mutation, which develop pancreatic ductal adenocarcinoma, it was reported that pancreatic EMT and dissemination precedes pancreatic tumor formation [10]. It would be possible to diagnose PanIN stage by *C. elegans* because in PKCY mice circulating tumor cells (CTC) were detected in 8 to 10-week old mice in PanIN stage. In this study, we did not assess the detection of CTC, Unresolved questions include whether diagnose using blood samples NSDT is highly economical diagnostic tool compared to traditional glycoprotein markers, such as CEA [17] and CA19-9 [17]. To detect cancer smells more precisely and quantitatively, it is necessary to identify specific cancer odours and their receptors. The reason why urine is suitable for decent detection has not been yet demonstrated.

## MATERIALS AND METHODS

### Mouse models of pancreatic cancer

Mice were maintained under specific-pathogen-free conditions and given free access to water and chow, under the ethical agreement of animal study in Osaka University (approved by professor Y. Kaneda as the approval number 24-122-022), of which experimental procedure had been performed as descried previously [14]. Briefly, Kras^LSL-G12D^ strain carries a loxP-stop-loxP sequence followed by constitutive active form of Kras^G12D^. Kras^LSL-G12D^ mice were obtained from the National Cancer Institute National Institutes of Health, Bethesda, MD, USA. Met^flox/flox^ transgenic mice from a BL6 background were obtained from the National Cancer Institute (National Institutes of Health, Bethesda, MD, USA) [15]. Pdx-1 promoter driven Cre^+/-^ mice, which express Cre specifically in pancreas were obtained from the National Cancer Institute National Institutes of Health, Bethesda, MD, USA. We have introduced Kras^G12D^ mutation in pancreas specific manner, by crossing Kras^LSL-G12D/+^ mice with Pdx-1-Cre^+/-^ mice. *Kras^LSL-G12D^* mice were crossbred to *c-Met^Fx^* to obtain mixed background (C57Bl/6/129/Sv) *Kras^LSL-G12D/+^;c-Met^Fx/Fx^* (for spontaneous tumors) or *Kras^+/+^,c-Met^+/+^* (control recipients) mice [14, 15]. We have generated mutation of Kras in pancreas specific manner in mice, which was compared with pancreas specific deletion of c-Met, as described [15]. In brief, pancreas specific transcription factor Pdx-1 driven Cre^/+^ mice, Kras^LSL-G12D/+^ mice and Met^flox/flox^ transgenic mating pairs of ~6 weeks old male and female mice from a BL6 background were obtained from the National Cancer Institute (National Institutes of Health, Bethesda, MD, USA) [15]. The Pdx-1Cre^/+^ mice were crossed with Kras^Lox-Stop-Lox(LSL)-G12D/+^ mice or Met^flox/flox^ mice to generate Pdx-1Cre^/+^/Kras^LSL-G12D/+^ and Pdx-1Cre^/+^/Met^flox/flox^ mice; both were crossed to generate double knockout, Pdx-1Cre^/+^/Kras^LSL-G12D/+^/Met^flox/flox^ mice. The genotypes were confirmed that alleles of Pdx-1Cre^/+^, KRAS^LSL-G12D/+^ and Met^flox/flox^ were present in the pancreases, but not tails, of compound mutant mice [15]. All experiments used co-housed littermates to ensure the consistency of microflora; the temperature was ~22° C in 12 h-cycle of light and dark, and food and water were added in adlibitum [15]. Samples such as urines was stored in freezer at −20° C until use.

### *C. elegans* cultures and strains

*C. elegans* strains were cultured at 20° C under standard conditions on Nematode Growth Medium (NGM) plates (1.7% Bacto agar, 0.5% Bacto peptone, 50 mM NaCl, 25 mM potassium phosphate buffer pH 6.0, 1 mM CaCl_2_, 1 mM MgSO_4_ and 5 μg/mL cholesterol) with *Escherichia coli* NA22, which grows in thick layers that serve as a suitable food source for large scale worm cultures used for chemotaxis analyses. The strain used in this study was wild type N2.

### Chemotaxis assays

The chemotaxis assays were performed using approximately synchronized young adult *C. elegans.* Grown nematode on NGM were collected in microfuge tubes and washed three times with basal buffer (5 mM potassium phosphate buffer pH 6.0, 1 mM CaCl_2_, 1 mM MgSO_4_ and 0.5 g/L gelatin). The nematode that settled at the bottom were taken and about 50–100 animals were spotted at the center of 9 cm assay plates (2.0% Bacto agar, 5 mM potassium phosphate buffer pH 6.0, 1 mM CaCl_2_, 1 mM MgSO_4_). 1 μL each of sample solution and 1 M sodium azide were spotted on two points separated by about 2.5 cm at one end of the plates. Only sodium azide was similarly spotted on the other side. The assays were conducted at the room temperature of 23 ± 1° C at 30 min. The chemotaxis index was calculated [12] (Figure 2) by using the formula, the chemotaxis index = (A − B)/ (A + B). Here, the side A was the number of animals on the sample-spotted side of the plate and B was the number of the animals on the opposite side, while animals that remained within 0.5 cm of the midline ignored to exclude immotile animals from consideration. Only well-fed nematodes were used in this study because starvation may affect their chemotactic responses.

### Sample collection

Urine samples were obtained by utilizing small pipet and 1.5 ml Eppendorf tubes as containers and on daytime. Approximately 1 ml urine was collected from each mouse, added to a polypropylene screw cap tube and stored at −20° C until use.

### Histopathological analysis

Pancreas from each mouse strain were removed and fixed with formalin. Paraffin-emended tissue sections were prepared as previously demonstrated [13, 14]. The specimens were then stained with hematoxylin and eosin. All sections were viewed with a Keyence BZ-9000 (Keyence Corp.).

### Statistical analysis

Differences in participants’ characteristics, laboratory data and tumour markers between the control and cancer participants were examined using the paired t-test for continuous variables and the χ2 test for dichotomized variables. A *P*-value of <0.05 was considered statistically significant.
